# Supplementary material for: MetaRibo-Seq measures translation in microbiomes
Source: Nat Commun. 2020 Jun 29;11:3268. doi: 10.1038/s41467-020-17081-z (PMC7324362; doi:10.1038/s41467-020-17081-z)
Supplement: Supplementary file 10 — Supplementary Data 7 [file 41467_2020_17081_MOESM10_ESM.zip › File2/Confidence_High_Taxonomy/93264_out.krona.html]

Javascript must be enabled to view this page.

members
magnitude
magnitudeUnassigned
count
unassigned
taxon
rank

93264\_out

2

2
2
superkingdom

phylum
1239
2

1
186801
class

186802
1
order

1898207
1

SRS043701\_contig\_number\_contig-100\_29253.29253
species

1
1262992
species

SRS149879\_contig\_number\_13125
